# Case Report: A Case Study Significance of the Reflective Parenting for the Child Development

**DOI:** 10.3389/fpsyg.2021.724996

**Published:** 2021-08-17

**Authors:** Zlatomira Kostova

**Affiliations:** Department of Psychology, Plovdiv University “Paisii Hilendarski, ” Plovdiv, Bulgaria

**Keywords:** traumatic experiences, emotional bonding, autistic spectrum disorder, family system, reflective parenting

## Abstract

There are studies that connect the “child” in the past with the “parent” in the present through the prism of high levels of stress, guilt, anxiety. This raises the question of the experiences and internal work patterns formed in childhood and developed through parenthood at a later stage. The article (case study) presents the quality of parental capacity of a family raising a child with an autism spectrum. The abilities of parents (the emphasis is on the mother) to recognize and differentiate the mental states of their non-verbal child are discussed. An analysis of the parental representations for the child and the parent–child relationship is developed. The parameters of reflective parenting are measured. The methodology provides good opportunities for identifying deficits in two aspects: parenting and the functioning of the child itself. Without their establishment, therapy could not have a clear perspective. An integrative approach for psychological support of the child and his family is presented: psychological work with the child on the main areas of functioning, in parallel with the therapy conducted with the parents and the mother, as the main caregiver. The changes for the described period are indicated, which are related to the improvement of the parental capacity in the mother and the progress in the therapy in the child. A prognosis for ongoing therapy is given, as well as topics that have arisen in the process of diagnostic procedures.

## Introduction

Attachment theory focuses on parent–child attachment and the effects this relationship has on the child's personality, interpersonal skills, and its capacity to form healthy relationships with adults. According to Bowlby (1969), parents who are approachable and responsive allow their child to develop a sense of security, thus creating a sound basis for it to learn about the world. The capability of parents to verbalize the feelings and experiences of their child through conversations, reading stories or fairy tales, commenting on everyday situations develops the skills of mentalization in the child (Ханчева, 2019).

In mature age, the ability to mentalize depends on the emotional load of the interpersonal situation. Optimal mentalization implies integration of cognitive knowledge with insights into the emotional world, which allows a man to see more clearly and achieve “emotional knowledge” (Allen and Fonagy, 2006).

The processes of mentalization can be influenced by the “heritage” that is passed down through generations. In their life experience, individuals operate and make their choices not being aware that they repeat the history of their ancestors. In part, these complex relationships can be seen, felt, or anticipated. They are experienced as elusive, insensible, unnamed, or secret, and may leave traumatic traces (Kellermann, 2001).

Tisseron (2011), associates the process of transmission of traumatic experience from generation to generation with three types of symbolization of experience: affective/sensory/motor, figurative, and verbal. If the event is symbolized in just one of the modalities, the results are associated with violation of mental life. The result becomes a distortion of the parent–child relationships, of their functioning.

The main psychopathological mechanisms that are activated in the transmission of mental content between individuals from generation to generation are associated with the identification and the projective identity. In this case of transmission through generations, insensible patterns, conflicts, scenarios and roles, ideals, and perceptions of the object are identified.

Children of severely traumatic parents reproduce scenes that their parents went through, trying to understand their pain, and at the same time establish a connection with them. They maintain family ties through the integration of parenting experiences. In the meantime, the parent seeks to teach his/her child survival strategies in situations of future persecution, thus passing on his/her traumatic experience (Baranowsky et al., 1998).

Wilgowicz (1999), introduces the term “vampire complex” describing the impact of unexpressed and insensible experiences passed down from generation to generation. These traumatic experiences form the unconscious connection between the generations which interferes the natural course of the processes of separation and individuation. This complex is associated with experience of the child who in its development turns out to be “locked” in the prison of the parental traumatic experience being neither alive, nor dead, or in other words, unborn.

Krystal (1978), describes the affective blindness of the principal caregiver as a characteristic of unprocessed traumatic experience (Den Velde, 1998; Коростелева et al., 2017). It is associated with incomplete integration of the somatic Self into the Self.

Ammon (2000), Hirsch (1994) describe in this context the “psychosomatic mother whose behavior is characterized by a lack of understanding of boundaries, intrusiveness, alexithymia, excessive concern for the physical functioning of her child, and at the same time “blind” to its psychic experiences.” Hope et al. (2019) report that maternal depression and complaints of psychological distress are associated with an increased risk of trauma and hospitalization for the age 3–11 years, with the highest being in the period 3–5 years. In another study, Baker et al. (2017), reported an increased risk of burns, poisoning, and fractures in children aged 0–4 years raised by depressed mothers and/or such found in an anxious episode. Postpartum depression in the mother presupposes a high risk of burns, fractures, poisoning (Nevriana et al., 2020).

The relationship between parental attitudes and child development is influenced by unconscious dynamics of the intrapsychic world of mother and father (Tagareva, 2019). The ability of parents for reflexion and metacognitive monitoring allows them to recognize and regulate, to modulate, to turn into a symbolic (verbal) form the states they observe in their child. This gives an opportunity to comprehend and return in an understandable form to the child interpretation of its state based on understanding and empathy. If this capacity fails, the parent cannot give an adequate and meaningful interpretation of what is happening, because he/she himself/herself gets lost and confused in his/her own (threatening his/her integrity) experiences, and strong, meaningless, overwhelming emotions. The consequences of the lack of a “secure base” in the face of the caregiver may be associated with: low self-esteem, behavior of decompensation under stress, inability to develop and maintain friendships, trust and intimacy, pessimism toward themselves, family, society (Matanova, 2015). The low level of reflexion on the trauma and the unaddressed traumatic experience as the mother's internal position, affect, and are a risk factor for, psychopathology later in the development.

In addition, parenting skills can be further tested when raising a child with Autism Spectrum Disorders in the family. Therapy for this nosology needs to include both psychological work with the child and support for the parents, especially for the mother, who in most cases limits her social roles and devotes herself only to parenthood. This is a serious argument to seek and optimize approaches in clinical practice to support the family environment in which children with neurodevelopmental disorders are raised.

## Materials and Methods

This article is designed to present a case of a family with a child diagnosed with Autism Spectrum Disorder, where the non-integrated individual traumatic experience in the mother (N.) affects the quality of her reflective parenting.

The analysis aims to display the status of individual functioning and skills for reflective parenting, as well as the effectiveness of psychological intervention to revive and optimize the relationship mother-child. Although the functioning of the mother is the focus of the present study, an analysis of parenting and the father has also been applied.

The study is a pilot one and marks the start of a project lasting over time.

Diagnostic tools have been used for:

- Assessment of the development and functioning of the child according to the methodology of Matanova et al. (Matanova and Todorova, 2013). The methodology includes research of cognitive, linguistic, social, emotional, and motor sphere of functioning. Based on the identified deficits, it is possible to arrange a therapeutic plan for the child.
- Self-assessment scales for the study of the quality of the parental relationship and the formed internal work patterns (of affection and romantic relationship) of N. with her parents:
  ° The Parental Reflective Functioning Questionnaire (PRFQ) by Luyten et al. (2017a,b). The PRF assessment screening tool provides additional evidence of the complexity and multidimensionality of the PRF (Luyten et al., 2009). It contains 18 items intended mainly for use in the study of PRF of parents with children aged 0–5 years. Three different aspects of PRF are evaluated on a 7-point Likert scale. Based on validated factor analysis, the authors identified three theoretically consistent and clinically significant factors, each of which included six items: (1) prementalization modes (PM), (2) certainty about mental states (CMS), (3) interest and curiosity about mental states (IC).
  ° Assessment of emotional bonding in the parent–child relationship (PBI) Gordon Parker (Parker, 1979; Parker et al., 1979). The questionnaire consists of two scales which measure the variables “Care” and “Overcare” or “Control” by evaluating basic parenting styles through the prism of children's perception. It consists of two identical questionnaires of 25 items, one for each parent.
- Family sociogram to report its representation in the current family.
  ° Version of Eidemiller and Cheremisin (Eidemiller et al., 2007). It is a drawing projective technique exploring several aspects: identify the position of the subject in the system of interpersonal relationships; determine the nature of communication in the family (direct or indirect). Dimensions: Number of family members who fall into the very circle; Size of the circles which mark the members; Disposition of circles (members) relative to each other (location); Distance between circles (members).

The case under study includes: demographic data of the family, anamnesis of the child (data obtained from psychological and medical research), prescribed therapy and progress, “The Time Line” (Stanton, 1992)—technique to retrieve significant events from the mother's history during the main stages of her development, located on the “axis of time,” data obtained from her psychological research—hers and her husband's.

## Results

N. is married with one child at 2.6 years, with suspected Autism Spectrum Disorder.

### Demographic Data at First Visit

Mother (N.)—age: 36 years, education: higher, occupation: technologist.

Father (K.)—age: 39 years, education: higher, occupation: technologist.

Now, the mother is taking care of her child. Only her husband works. They live alone in a small town. The child is separated in his own room.

The child—bears his father's first name. According to parents: does not speak, does not eat independently—“He opens his mouth a little,” walks on tiptoe, does not play with other children, does not obey to commands, gets tired easily. The child attends the nursery until noon (on the recommendation of the director of the institution: “He does not eat”) and the Municipal Center for Personal Development. A social pedagogue works with him.

### Data for Assessment of the Child's Development

The child was carried to full-term, born from a second, pathological pregnancy of the mother, laid in bed to avoid miscarriage in the first months. He had a protracted jaundice, which passed after a year and a half. He was not breastfed.

After a consultation with a psychologist, dysfunction was found in the following areas: Sensory: the child does not hold pelvic reservoirs, shows behavior of sensory hunger—needs intensely sensory stimuli; *Motor development:* with evidence of late walking, the child steps on toes; *Cognitive processes:* the child has not yet formed a body schema, he tends to suck the thumbs of his lower limbs; he still explores the objective world through oral modality; passivity regarding the choice of a toy if it is not in his filed vision; he does not play with his toys as intended; *Emotional and social functioning:* he is easily separated from the adult; the emotional expression is poorly differentiated and is played through the body by waving hands; lack of social interest; interaction is possible after prolonged sensory stimulation. *Language development:* he vocalizes; does not respond to his name.

During the study, the child is calm, passive. When coming into interaction, he retains his interest in the adult, but without any initiative to develop it further.

Electroencephalography was performed, in awake state and with open eyes, which displayed mixed main activity: of diffuse beta waves, and tetha waves 4.5–5 Hz, in the anterior areas: sporadically slower waves 3–4 Hz.

The child was prescribed a therapy with psychologist with live setting twice a week. The therapy with the parents was once a week. It started online prior to the beginning of the therapy with the child due to COVID-19 quarantine. Twenty sessions were held with the child, i.e., work continued for 5 weeks (with setting twice a week). The therapy includes psychological work with the child in the main areas of functioning, established as therapeutic lines of the conducted diagnostics. Ten sessions were held with the parents and the mother. Two of the sessions were held with the parents. The following were studied: their functioning through the different subsystems: marital, parental, child–parental; difficulties in raising a child with an autism spectrum. It was found that the family system organized its resource for therapy only for the child. They realized that their well-being was important for their child's development. The marital subsystem was in the background. A session was held with the father, in which his role as the Third Significant in the child's life was discussed. Seven sessions were held with the mother. In them was unfolded her personal story through early experience, child–parent relationship, main topics of growing up, intimacy, parent–child relationship with her child. The therapy is going on.

### Progress of the Therapy With the Child

Decrease of sensory hunger, no tactile simulation is required to activate the child to study the objective reality; *General motor skills:* reduced toe walking, except in moments of agitation, he walks on a full step on a sensory path. The child jumps on tiptoe, climbing stairs is easier than getting down; *Fine motor skills:* improved grip (small toys, sticks, without clenching them in the fist); *Cognition:* recognizes himself in the mirror, experiments on dropping toys (primary circular reactions). Still uses oral inspection of some toys, beginnings of a play by designation (zone of proximal development). The active choice of toys is in progress, he explores freely the specialist room. Object constancy is formed, he seeks an object which he has played with. Lively, interesting. *Emotional development:* he expresses his joy by shouting and laughing, rejoices when imitated. Expresses anger. Attempts to manipulate by imitating crying. *Language development:* sporadically pronounces syllables, still does not respond to his name; *Peculiarities:* likes objects with small holes and pays lasting attention to them. He enters the oral-sadistic stage, bites toys, and gnaws some of them. Learned helplessness.

**Table:** Time line of N.

*Facts from family history:*	The maternal grandfather: violence, anti-social behavior, alcohol, suicide; The mother: eyewitness of domestic violence; parenting to her younger brother. Lack of communication with the family of her father. Born in a two-parent family, after attempts of many years of her mother to get pregnant. She has a senior brother, who was adopted.
*Childhood*	*About her mother:* Her memories of her mother are associated with neglect, emotional alienation, austerity. According to her mother, N. was a meek child, she did not create any “problems.” She talks about her in anger. “No hugs, no kisses. There were no songs, no fairy tales.”; “My mother meddled into everything.”; “She was calling my friends.”; “She talked to my female teachers when my first cycle appeared in third grade.” Shame; “She was strutting with in front of the whole village.”; “Because of you, your father passed away.” Guilt. Her mother was trying to impose her own pattern of behavior. For this reason, now that N. falls in an unfamiliar situation, she feels insecure and embarrassed.
	*About her father:* According to her, her father was the warmer person, emotionally. She did not receive any praise from him either: “He did it silently.” There are no specific memories of him, as well as no connection with any paternal relatives. “He was very calm and supporting.” She refers to the age of 4–5 years. It is difficult for her to follow the chronology of her experiences.
*Adolescence*	*Self-identity:* heavy menstrual cycle. *Experiencing shame* before the teachers and the class from her first menstruation, after her mother's intervention. *Friendships:* her mother interferes in her choices. She was forced to make choices impossible for her life experience on behalf of her mother (abortion of the mother).
*Romantic relationship*	6 years of unrequited love, before her husband. Her mother again interfered in her choices.
*Parenting*	Half a year after a suffered miscarriage she became pregnant again. She refused to breastfeed her child: “I had no desire to breastfeed him. I can't imagine this connection between us.” Her mother insisted on breastfeeding, but she refused in protest. Through the unprocessed mourning, she presented herself as an overprotective parent. The child began to walk later. He has been protected from climbing stairs for a long time. As a result, he did not develop his motor skills. His eating was difficult and selective: “he refuses to chew but chews a banana.” He was fed in front of the TV. Eye contact was unstable. N. tried to remove the diaper by placing his pot among the toys during play and urged the child: “Come on, pee!.” She put him to sleep after a long intense swing. There was no established daily routine. “My child does not want anything. He has no desires. It is my fault that my child is like that.” That was her motivation to start therapeutic work for her. *She describes the emotional connection with her child as:* “Strong” —“it is difficult to fall asleep without my presence”; “Joy”—“when he sees me, he comes to hug me”; “He associates me with eating”—he turns to me more often when he is hungry—mainly I feed him. So far no one else has fed him. When he sees me, he says “Am-am” *Wishes for the future of her child:* “To have a good education—to choose for himself. Let his choice be important, not we to…”; “To have good friends around him” “To have the quality to judge people—what is good for him, what is wrong. To be able to sift through the good”; To be safe and sound.” “if it can be sports, to dance, no matter. He is very attracted to dancing: dance, music, mathematics.”
*Personality*	*Phobic*—*iatrophobia/*castration anxiety (fear of body damage, especially sexual mutilation)—*ashamed to go to a gynecologist (at the age of 26 she visited a gynecologist for the first time due to reproductive dysfunctions), separation anxiety: “I always held her hand (my mother's),” social anxiety; “People are not what I imagine them to be.” Distrust, suspicion*. *Traumatic*—*She has not freed herself from the trauma in the past: “Looking at him, how timid and insecure he is, as if I were looking at you as a child.” “I shivered.”*
*Medical history*	at birth, “hormonal crisis of the newborn”—(*vaginal bleeding due to certain endometrial hyperplasia as a result of the intrauterine exposure to maternal estrogens and the subsequent postpartum fall of their levels*). at the age of 4 fracture of the left leg; at the age of 13 cracked beard from falling; at the of 10 “I struck dumb” from poor grades, she was forced to call her stepfather “dad”; “fainting-fits” at the age of 13, 14, 29 in public places. *Presently*: migraine pain, tightness in the throat, impaired hormonal balance in the initial stage (she takes L-Thyroxyn).
*Losses:*	*The father* at the age of four. She had a severe fracture of her left leg, and as she recollected, she was sitting alone on a carpet in an apartment when he “died outside.” Her older brother, who had been adopted, informed her of the loss, forbidding her to speak because he was threatened by their mother that if he revealed the secret, she would be angry with him. She had to be hospitalized for 20 days while her mother and brother handled the transportation of the body and the funeral of her father in Bulgaria (the event occurred outside the country). The reason for the absence of her relatives was not explained to her. *Natural abortion*. After many years of long attempts to conceive, she lost her fetus in the fifth month (“They saved my life instead of the child's life.”) The two parents were incompatible. Her mother stated: “You will not get pregnant! You will bring a sick child into the family.”

### Data From Performed Psychological Studies

#### Child–Parent Relationships and Internal Work Patterns for Oneself and for the Other (PBI)

The results of the self-assessment questionnaire on emotional closeness in the parent–child relationship with the mother indicate:

With reference to the relations with her mother: high results along the dimension “Overcare/Control” (24 points) and low results along the dimension “Care/Concern” (22 points). From these results it is evident that the mother in childhood is represented as emotionally cold, indifferent, and careless, and at the same time imposing control, intrusiveness, and excessive contact, infantilizing, and hindering the autonomy of N. as a child.
With reference to the relations with her father: high results along both dimensions “Overcare/Control” (32 points) and “Care/Concern” (25 points) what relates to a representation of the father's character as emotionally restrained in his behavior, but at the same time controlling, intrusive, and in attitude which is highly infantilizing and hindering the autonomy of N.

The model of adult attachment, proposed by Bartholomew and Horowitz (1991), related through the Parker quadrant, for the emotional closeness of a child–parent shows that N. has an active negative internal work pattern for herself along the dimension of “anxiety” and is associated with vulnerability to separation, rejection, or insufficient love. The work pattern of the other is negative, associated with fear of intimacy and social avoidance, i.e., along the dimension of “avoidance.” The attachment style corresponds to style B avoidant, subcategory cowardly avoidant.

In her husband, the internal work pattern is ambivalent. The mother's character from childhood is represented as emotionally restrained and controlling, while the father's is emotionally indifferent, however encouraging autonomy.

#### Family Sociogram

As a child, she presented inadequate, low self-esteem, and anxiety, an experience of emotional rejection and isolation. The father was the most significant figure, he was more emotionally close. This is also observed in her relations with the maternal grandmother. The size and thickening of the circle, which it is represented with, shows high levels of intrapersonal neuroticism. There are too many figures in the circle: apart from her four-member family, mother, father, brother, and she, it also includes her maternal grandmother and her uncle, the brother of her mother, as the division is in two camps on the basis of proximity-distance: her mother, uncle, and brother are found at one end of the circle, and the other end is occupied by her, her father and grandmother.

As an adult, prior to the birth of her child, her mother was also included in the circle which is associated with a tendency to unsatisfied needs from her.

After the birth of the child, the hierarchy is maintained and there is enough space between the members of the family now.

Through the life cycle of the family and the separation/individuation, this crisis must be lived through and integrated as a new experience. The stages show that in her childhood N. did not have a sound family model, the boundaries between the parent family and the maternal family are permeable. The above configuration could be interpreted as the presence of triangulations in the family system, and as well as intergenerational ones.

Within the romantic couple, in the period of the dyad, N. presents herself and her husband in a line, as the lower part of the test field includes the figure of her mother depicted by a smaller circle. This could be interpreted with the still insufficient density of the family boundaries. Establishing family boundaries (internal and external) is an important task at this stage of family life, as well as creating an optimal balance of proximity and distance; distribution of the roles in the family; establishing the hierarchy; negotiating family rules; coordination of future life plans, as well as joint understanding and acceptance.

It is also confirmed by the results of the interpretation of the family sociogram with the father as well. As a child he presented himself with inadequate, low self-assessment, he was hierarchically placed next to the mother's figure. Prior to the birth of the child, he presented unsatisfied needs from his parental family: no separation, the boundaries between own and native family are permeable. In the present one, the experience in the reality of what is happening is available. There is no differentiation between the relationships, and dissatisfaction with them is present. The child is put in the place of unsolved contradictions.

#### Reflective Parenting PRFQ

In all three dimensions, the results show values above the average as IC (“interest and curiosity about mental states”) is leading-−85.5%. It is associated with intrusive hypermentalization, i.e., she is difficult to regulate and interpret her own mental states when faced with her unregulated, difficult child. As a sequence, an inadequate reaction in response to his affective signals by the mother is provoked, as well as the presence of low levels distress tolerance. In hypermentalization as a process, there is a tendency to understand or explain mental dynamics based on complex logical constructs, sometimes abstract, notional, and without pragmatic benefit. Its extreme forms are characterized by autistic, groundless fantasizing.

The possibilities for reflective parenting with the father show increased trends in the dimensions of IC (“interest and curiosity about mental states”) and CMS, which is associated with enhanced hypermentalization, as in the mother, in the cases when she does not recognize the vague mental states of her child, however, here is also a desire to understand.

## Discussion

### Mother

In her story N. unfolds a picture of the transmission of a traumatic experience of rejection/avoidance. The experience of emotional neglect has formed a negative notion of the Self. Through her anger, she repeats the model of her mother, not realizing that her own model is possible.

N. demonstrates a personal style in which fear and anxiety constitute a centrally organizing dimension. Reported phobias are associated with behaviors of shyness, restraint, aptitude for low self-esteem, indecisiveness, uselessness, and emotional inhibition. It is difficult for her to **identify** anxious thoughts, as well as to **connect** them with their triggers from reality, to **master** them and to allow a **“decentralized”** point of view on anxious situations, what might be the birth and upbringing of a child with arrested development. Avoidance behavior is associated with a remarkably high level of distress and a low level of long-term adaptation. (Mikulincer and Shaver, 2012; Lingiardi and McWilliams, 2017). In cognitive theory, this feature (functioning through fear and avoidance) is considered an excellent example of an early maladaptive self-assessment scheme. The theory of mentalization conceptualizes this as an implicit (automatic) mentalizing deficit. In addition, there are difficulties in understanding the mind of others (Dimaggio et al., 2007; Lampe and Malhi, 2018). Another major deficit of mentalization is their weak affective consciousness (Steinmair et al., 2020).

### Mother–Child Relationship

The relationship with her child is not objective. There is no construct to include references to the related problems outlined in her child. N. includes projective identification against guild as a protective mechanism related to her wishes for the child's future. The relationship with her child is idealized, in her aspiration and strong desire for love, characteristic of her personal structure. In this case, the child serves the mother's deficits and is not perceived objectively. The projection also supports this structure in her fear of rejection. She is parenting by satisfying the child's physical needs without giving the father the opportunity to be introduced to the child's mental life. And, although the projection is central to the father, in describing the relationship with the child, their shared experiences are related to “curiosity,” “play.” The mother's fear of loss, of rejection is the result of the unprocessed mourning. It could be also thought of splitting through the non-integrated image of the early figure of attachment. Presently, she is still demonized, and the father is idealized.

### The Child

In the described period the child's study of objective reality is activated. recognizable in a mirror. Demonstrates the beginning of a game as intended, expresses joy in interaction, anger. Attempts to manipulate through imitation.

### Parent Couple

The possibilities for reflective parenting in both parents are associated with increased hypermentalization, and the father has a desire to understand the mental states of the child.

### Married Couple

N.'s internal working models are of a cowardly avoidant style (her husband's internal working model is ambivalent). The level of adherence to therapy is low, a high level of symptom reporting, and a low level of basic confidence. Those who have a negative BPM for themselves and for the other both want and fear of intimacy in the couple. This also presupposes the future occurrence of crisis in N. married couple.

### Family System

In families such as the above described, raising a child who is unable to express their own needs in a conventional way, unresolved conflicts from the beginning of their life cycle, can escalate and lead to marital dissatisfaction and dysfunction throughout the family system.

## Conclusion

The presented integrative model of psychological support in a family raising a child with an autistic spectrum outlines a picture of improvement in two lines: in the child and in the child–parent relationship. In mother, the process of disidentification, the formation of the transmission of the object, the separation of what has been transmitted to it, allows the history of the past to be restored, therefore gives more freedom to the individual in the shaping of the individuality. Currently, the inserted traumas, even if not one's own, in the subjective experience of conflicts and fantasies, allow to integrate this experience and to turn it from destructive to structuring.

If the traumatic event is mentally processed, symbolized, and inserted in the individual memory as an experience, it receives the status of the past, of memory. It is passed on to generations not only as the content of traumatic experience but also the aptitude of its mental processing and coping with it, which affects the individual development of the child.

N.'s feedback on the therapy so far: “He showed it to us, but I, my fault, my mistake, was that I did not see it.” She finds that now is more observant.

## Data Availability Statement

The datasets generated for this article are not readily available because personal data. Requests to access the datasets should be directed to Zlatomira Kostova.

## Ethics Statement

Ethical review and approval was not required for the study on human participants in accordance with the local legislation and institutional requirements. Written informed consent to participate in this study was provided by the participants' legal guardian/next of kin. Written informed consent was obtained from the individual(s), and minor(s)' legal guardian/next of kin, for the publication of any potentially identifiable images or data included in this article.

## Author's Note

The article presents a research perspective on the possibilities of parental capacity, through the integration of different approaches to understanding human suffering in clinical psychology.

## Author Contributions

The author confirms being the sole contributor of this work and has approved it for publication.

## Conflict of Interest

The author declares that the research was conducted in the absence of any commercial or financial relationships that could be construed as a potential conflict of interest.

## Publisher's Note

All claims expressed in this article are solely those of the authors and do not necessarily represent those of their affiliated organizations, or those of the publisher, the editors and the reviewers. Any product that may be evaluated in this article, or claim that may be made by its manufacturer, is not guaranteed or endorsed by the publisher.

## References

[B1] Коростелева И. С. Ульник Х. Кудрявцева A. В. Pатнер Е. A. (2017). Трансгенерационная передача: роль трансмиссионного объекта в формировании психосоматического симптома. Журнал практической Психологии Психоанализа.

[B2] Ханчева К. (2019). Mентализация и ранни етапи на социо-емоционално развитие. Университетско издателство. Климент Охридски.

[B3] Allen J. G. Fonagy P. (eds.). (2006). The Handbook of Mentalization-Based Treatment. Hoboken, NJ: John Wiley and Sons. 10.1002/9780470712986

[B4] Ammon G. (2000). Psychosomatic Therapy. Saint Petersburg, FL: Speech.

[B5] Baker R. Kendrick D. Tata L. J. Orton E. (2017). Association between maternal depression and anxiety episodes and rates of childhood injuries: a cohort study from England. Inj. Prev. 23, 396–402. 10.1136/injuryprev-2016-042294 28232401

[B6] Baranowsky A. B. Young M. Johnson-Douglas S. Williams-Keeler L. McCarrey M. (1998). PTSD transmission: a review of secondary traumatization in Holocaust survivor families. Canad. Psychol. 39:247–256. 10.1037/h0086816

[B7] Bartholomew K. Horowitz L. M. (1991). Attachment styles among young adults: a test of a four-category model. J. Pers. Soc. Psychol. 61, 226–244. 10.1037/0022-3514.61.2.226 1920064

[B8] Bowlby J. (1969). Attachment and Loss. v. 3, Vol. 1. New York, NY: Basic Books.

[B9] Den Velde W. O. (1998). Children of Dutch war sailors and civilian resistance veterans, in International Handbook of Multigenerational Legacies of Trauma, ed Danieli Y. (Boston, MA: Springer), 147–161. 10.1007/978-1-4757-5567-1_10

[B10] Dimaggio G. Semerari A. Carcione A. Nicolo G. Procacci M. (2007). Psychotherapy of Personality Disorders: Metacognition, States of Mind and Interpersonal Cycles. London: Routledge. 10.4324/9780203939536

[B11] Eidemiller E. G. Alexandrova N. V. Justickis V. (2007). Family Psychotherapy. Saint Petersburg, FL: Speech.

[B12] Hirsch M. (1994). The body as a transitional object. Psychother. Psychosom. 62, 78–81. 10.1159/000288907 7984771

[B13] Hope S. Pearce A. Chittleborough C. Deighton J. Maika A. Micali N.. (2019). Temporal effects of maternal psychological distress on child mental health problems at ages 3, 5, 7 and 11: analysis from the UK Millennium Cohort Study. Psychol. Med. 49, 664–674. 10.1017/S0033291718001368 29886852 PMC6378410

[B14] Kellermann N. P. (2001). Transmission of Holocaust trauma-an integrative view. Psychiatry 64, 256–267. 10.1521/psyc.64.3.256.18464 11708051

[B15] Krystal H. (1978). Trauma and affects. Psychoanal. Study Child. 33:81–116. 10.1080/00797308.1978.11822973 715118

[B16] Lampe L. Malhi G. S. (2018). Avoidant personality disorder: current insights. Psychol. Res. Behav. Manag. 11, 55–66. 10.2147/PRBM.S121073 29563846 PMC5848673

[B17] Lingiardi V. McWilliams N. (eds.). (2017). Psychodynamic Diagnostic Manual: PDM-2. New York, NY: Guilford Publications. 10.4324/9780429447129-11

[B18] Luyten P. Fonagy P. Mayes L. Van Houdenhove B. (2009). Mentalization as a multidimensional concept. Manuscript submitted for publication.

[B19] Luyten P. Mayes L. C. Nijssens L. Fonagy P. (2017a). The parental reflective functioning questionnaire: development and preliminary validation. PLoS ONE 12:e0176218. 10.1371/journal.pone.0176218 28472162 PMC5417431

[B20] Luyten P. Nijssens L. Fonagy P. Mayes L. C. (2017b). Parental reflective functioning: Theory, research, and clinical applications. Psychoanal. Study Child. 70, 174–199. 10.1080/00797308.2016.1277901

[B21] Matanova V. (2015). Attachment: There and Then, Here and Now. Varna: Steno.

[B22] Matanova V. Todorova E. (2013). Guide for Applying a Methodology for Assessing the Educational Needs of Children and Students. Institute of Mental Health and Development.

[B23] Mikulincer M. Shaver P. R. (2012). An attachment perspective on psychopathology. World Psychiatry 11, 11–15. 10.1016/j.wpsyc.2012.01.003 22294997 PMC3266769

[B24] Nevriana A. Pierce M. Dalman C. Wicks S. Hasselberg M. Hope H.. (2020). Association between maternal and paternal mental illness and risk of injuries in children and adolescents: nationwide register based cohort study in Sweden. BMJ 369:m853. 10.1136/bmj.m853 32269017 PMC7190076

[B25] Parker G. (1979). Parental characteristics in relation to depressive disorders. Br. J. Psychiatry 134, 138–147. 10.1192/bjp.134.2.138 427329

[B26] Parker G. Tupling H. Brown L. B. (1979). A parental bonding instrument. Br. J. Med. Psychol. 52, 1–10. 10.1111/j.2044-8341.1979.tb02487.x

[B27] Stanton M. D. (1992). The time line and the “Why now?” Question: a technique and rationale for therapy, training, organization consultations and research. J. Marit. Fam. Ther. 18, 331–343. 10.1111/j.1752-0606.1992.tb00947.x

[B28] Steinmair D. Richter F. Loeffler-Stastka H. (2020). Relationship between mentalizing and working conditions in health care. Int. J. Environ. Res. Public Health. 17:2420. 10.3390/ijerph17072420 32252375 PMC7178150

[B29] Tagareva K. (2019). Psychological readiness for motherhood in pregnant women in different social environments, in Proceedings of the Interdisciplinary Scientific Conference of the Faculty of Pedagogy at Plovdiv University: “Man and Global Society.” (Plovdiv).

[B30] Tisseron S. (2011). Secrets de Famille Mode D'emploi. Paris: Marabout. 10.3917/puf.tisse.2011.02

[B31] Wilgowicz P. (1999). Listening psychoanalytically to the Shoah half a century on. Int. J. Psychoanal. 80, 429–438. 10.1516/0020757991598765 10407742

